# STIM and Orai1 Variants in Store-Operated Calcium Entry

**DOI:** 10.3389/fphar.2015.00325

**Published:** 2016-01-13

**Authors:** Juan A. Rosado, Raquel Diez, Tarik Smani, Isaac Jardín

**Affiliations:** ^1^Department of Physiology, Cell Physiology Research Group, University of ExtremaduraCáceres, Spain; ^2^Department of Medical Physiology and Biophysic, Institute of Biomedicine of Seville, Virgen del Rocio University Hospital, Consejo Superior de Investigaciones Científicas, University of SevilleSevilla, Spain

**Keywords:** calcium entry, STIM1, STIM2, orai1, splice variants

## Abstract

Store-operated Ca^2+^ entry (SOCE) is an ubiquitous mechanism for Ca^2+^ entry in eukaryotic cells. This route for Ca^2+^ influx is regulated by the filling state of the intracellular Ca^2+^ stores communicated to the plasma membrane channels by the proteins of the Stromal Interaction Molecule (STIM) family, STIM1, and STIM2. Store-dependent, STIM1-modulated, channels include the Ca^2+^ release-activated Ca^2+^ channels, comprised of subunits of Orai proteins, as well as the store-operated Ca^2+^ (SOC) channels, involving Orai1, and members of the canonical transient receptor potential family of proteins. Recent studies have revealed the expression of splice variants of STIM1, STIM2, and Orai1 in different cell types. While certain variants are ubiquitously expressed, others, such as STIM1L, show a more restricted expression. The splice variants for STIM and Orai1 proteins exhibit significant functional differences and reveal that alternative splicing enhance the functional diversity of *STIM1*, *STIM2*, and *Orai1* genes to modulate the dynamics of Ca^2+^ signals.

## Introduction

Eukaryotic cells finely modulate cytosolic calcium concentration ([Ca^2+^]_c_) to trigger a myriad of physiological events, from short term responses, such muscle contraction, impulse transmission, secretion, and aggregation, to long term events, including activation of transcription factors, growth and in the last instance, apoptosis, and cellular death. Evolution has provided the cells with a highly complex machinery, which finely tunes, orchestrates and coordinates intracellular Ca^2+^ homeostasis. Physiological agonists modulates [Ca^2+^]_c_ by the activation of more or less selective Ca^2+^ channels and transporters, both in the intracellular Ca^2+^ compartments [endoplasmic reticulum (ER), mitochondria or acidic vesicles] and in the plasma membrane (PM). Once the stimulus ends, [Ca^2+^]_c_ returns to basal level and the cell is ready for a new stimulation (Berridge et al., 2003; Redondo and Rosado, 2015). Cells have a number of mechanisms to induce Ca^2+^ entry and although all these events were studied in the past as independent pathways, mostly due to technical challenges to address Ca^2+^ signal as a whole, new studies and a deeper comprehension of Ca^2+^ entry support a sophisticated relation encompassed by all these pathways (Mignen et al., 2007; Wang et al., 2010; Rodriguez-Moyano et al., 2013; Zhang et al., 2014).

Store-Operated Calcium Entry (SOCE), a major mechanism for Ca^2+^ influx, is regulated by the filling state of the intracellular Ca^2+^ reservoirs, mainly the ER. A reduction in the intraluminal ER Ca^2+^ concentration ([Ca^2+^]_ER_), evokes the opening of channels in the PM leading to Ca^2+^ entry from the extracellular medium (Putney, 1986). After intense investigation, the mechanism that communicate the filling state of the intracellular Ca^2+^ stores to the PM channels was found to be mediated by the Stromal Interaction Molecule 1 (STIM1), a protein discovered in Oritani and Kincade (1996) and known as a cell–cell interaction mediator. STIM1 is the ER Ca^2+^ sensor that stimulate Ca^2+^ entry, triggering the activation of store-operated channels located in the PM (Roos et al., 2005; Zhang et al., 2005). Concerning the Ca^2+^-permeable channels that conduct SOCE, soon after the identification of STIM1 as the ER Ca^2+^ sensor Orai1 was proposed as the pore-forming subunit of the Ca^2+^ release-activated Ca^2+^ (CRAC) channels (Feske et al., 2005, 2006; Mercer et al., 2006; Peinelt et al., 2006; Prakriya et al., 2006). In addition, STIM1 might activate the less Ca^2+^ selective store-operated Ca^2+^ (SOC) channels, which require the interaction of Orai1 with the canonical transient receptor potential (TRP) family member TRPC1 (Rosado and Sage, 2000; Singh et al., 2000; Huang et al., 2006; Yuan et al., 2007; Jardin et al., 2008; Cheng et al., 2011, 2013; Choi et al., 2014; Desai et al., 2015). STIM1 activates Orai1 through a cytosolic STIM1-Orai1 activation region (SOAR; aa 344–442; Yuan et al., 2009) also identified as the CRAC activating domain (CAD; aa 342–448; Park et al., 2009); the Orai-activating small fragment (OASF; aa 233–450/474; Muik et al., 2009) and the Ccb9 (aa 339–44; Kawasaki et al., 2009). SOAR dimerization is essential for the activation of Orai1 and the polybasic region (aa 382–387) within the SOAR structure is required for Orai1 binding (Yang et al., 2012). The activation of TRPC1 by STIM1 has been reported to require both, the SOAR region, which is important for the STIM1–TRPC1 interaction (Lee et al., 2014), and the last 14 amino acids of STIM1, which constitute a polybasic lysine-rich domain required for the activation of TRPC channels by STIM1 upon store depletion (Zeng et al., 2008).

Furthermore, Orai1, together with Orai3, and the PM-resident STIM1 have also been reported to participate in a store-independent mechanism for Ca^2+^ entry activated by arachidonate (Mignen et al., 2008a,b, 2009), which reveals the diversity and complexity of the regulation of Ca^2+^ entry in eukaryotic cells.

## STIM Proteins

**FIGURE 1:**
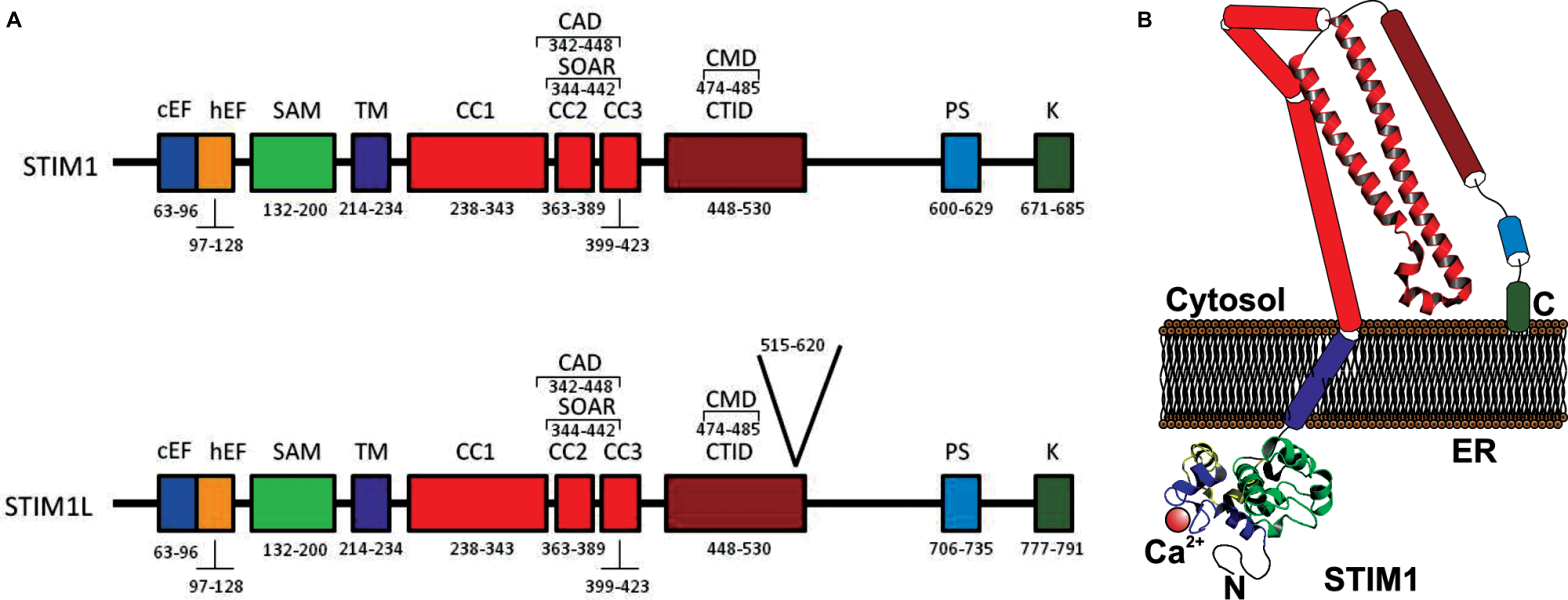
**(A)** Molecular structure of STIM1 and STIM1L. **(A)** The ER luminal N-terminal region includes a Ca^2+^-binding canonical EF-hand motif (cEF), a hidden EF-hand (hEF) motif, and a sterile α-motif (SAM). STIM1 has a single transmembrane domain^TM^. The cytosolic C-terminal region includes three coiled-coil (CC) regions (CC1, CC2, and CC3), which contain the SOAR (STIM–Orai activating region) or CAD (CRAC activation domain), the minimal sequence required for the activation of Orai1 channels. Downstream of CC3 there is a C-terminal inhibitory domain (CTID), which overlaps the CRAC modulatory domain. The C-terminal region also contains a Pro/Ser-rich domain (PS) and a Lys-rich domain (K) (Soboloff et al., 2012). STIM1L also includes 106 amino acids located a position 515–620 (Darbellay et al., 2011). **(B)** Cartoon depicting a possible model of STIM1 monomer in the coalescent state, where STIM1 solved crystal structures are shown. CC1 is divided in CC1α1, CC1α2, and CC1α3 (Fahrner et al., 2014).

**FIGURE 2:**
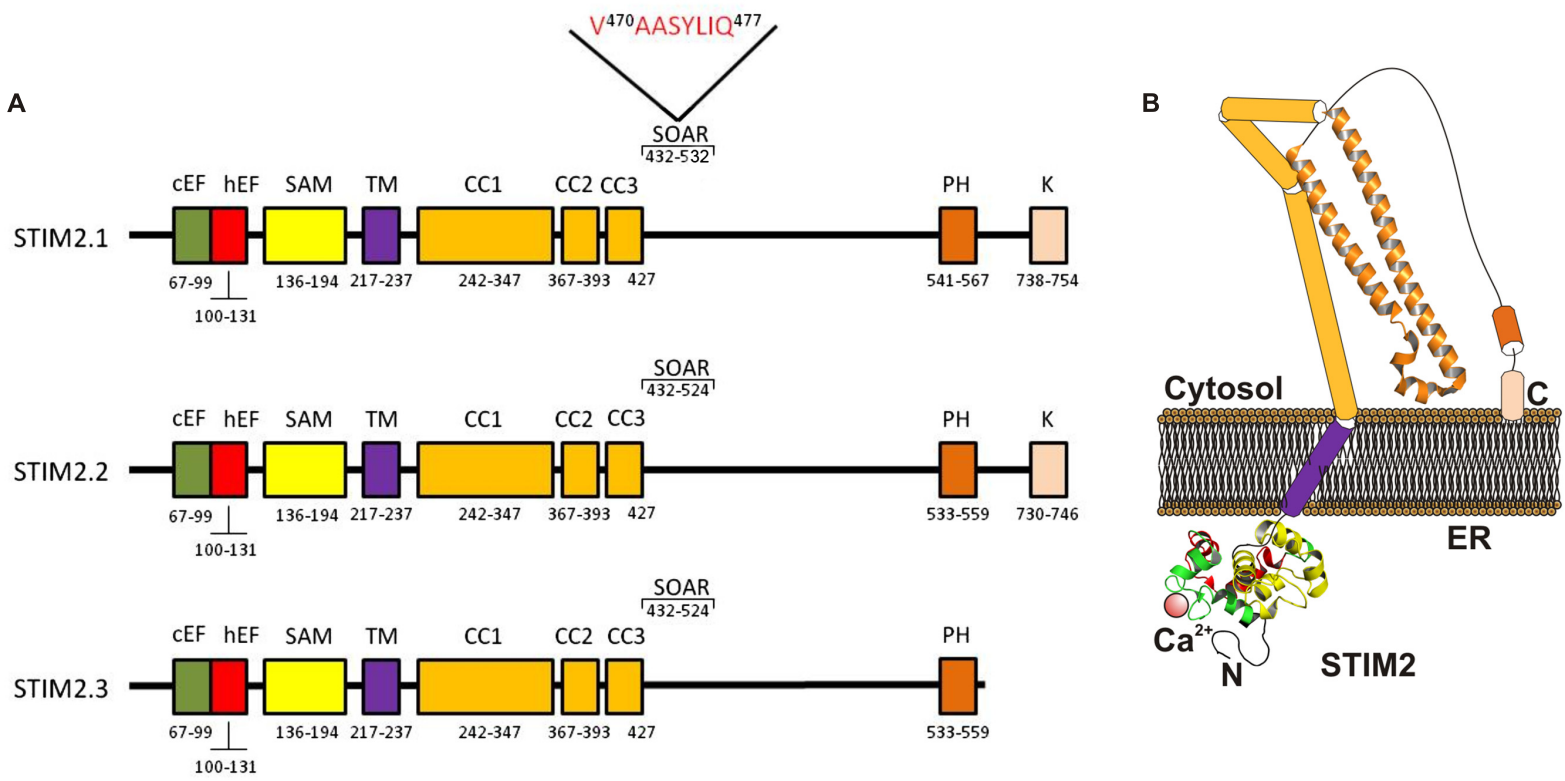
**Molecular structure of STIM2 variants. (A)** The ER luminal N-terminal region includes a Ca^2+^-binding canonical EF-hand motif (cEF), a non-Ca^2+^ -binding hidden EF-hand hEF motif, and a sterile α-motif (SAM). The cytosolic C-terminal region includes the CC regions (CC1 and CC2), the SOAR region a Pro/His-rich (PH) and a Lys-rich domain (K). The STIM2.1 variant contains an eight-residue insert (VAASYLIQ) in its SOAR region (Miederer et al., 2015; Rana et al., 2015). **(B)** Cartoon depicting a possible model of STIM2.2 monomer in the coalescent state, where STIM1 solved crystal structures conserved within STIM2 are shown. CC1 is divided in CC1α1, CC1α2, and CC1α3 (Fahrner et al., 2014).

Members of the STIM family, STIM1 (**Figure 1**) and STIM2 (**Figure 2**), have highly conserved structure and present slightly divergences giving them different functions. Upon store depletion, STIM1 oligomerizes and redistributes into discrete puncture nearby the PM (Luik et al., 2008; Cahalan, 2009; Park et al., 2009; Covington et al., 2010).

Both STIMs are single spanning transmembrane (TM) proteins that are located mainly in the ER (Roos et al., 2005; Zhang et al., 2005; Baba et al., 2006), but also in acidic stores (Zbidi et al., 2011) and in the PM (Sabbioni et al., 1999; Spassova et al., 2006; Jardin et al., 2013). The STIM N-terminal region is located in the intraluminal compartment (or the extracellular medium when located in the PM), harboring the canonical and hidden EF-hand (hEF) motives (for STIM1 aa 63–128; Liou et al., 2005; Roos et al., 2005). The Ca^2+^ binding canonical EF-hand is the Ca^2+^ sensor. Mutations within this region incapacitate Ca^2+^ association, thus, inducing constitutive Ca^2+^ entry (Spassova et al., 2006). Next, STIM presents the steril-α-motif (SAM) domain (aa 132–200) exhibiting distinct properties in STIM1 and STIM2 (Stathopulos et al., 2009; Zheng et al., 2011). SAM is followed by the TM domain (aa 214–343), which has been recently shown to undergo structural changes from the resting state, where Ca^2+^ is bond to the EF-hand, to the activated one, when store depletion occurs (Ma et al., 2015). Located in the cytosol, STIM C-terminus comprises 3 conserved coiled-coil (CC) domains, CC1 (aa 238–343), CC2 (aa 363–389), and CC3 (aa 363–389), the CRAC modulatory domain (CMD; aa 448–530), which includes the STIM1 homomerization domain SHD (aa 420–450), followed by a serine/proline rich region (aa 600–629) and a lysine-rich region (aa 671–685) at the very end of the C-terminus, that binds to membrane phospholipids, thus anchoring STIM1 toward its target (Liou et al., 2005). Furthermore, the polybasic lysine-rich region regulates Ca^2+^ entry by PM-resident STIM1 (Jardin et al., 2009, 2013). The long CC1 domain might be separated into CC1_α1_, CC1_α2_, and CC1_α3_ (Soboloff et al., 2012; Yang et al., 2012; Stathopulos et al., 2013). Furthermore, CC2 and CC3 domains, which comprise the SOAR domain, could be divided in four regions, Sα1, Sα2, Sα3, and Sα4 (Yang et al., 2012; Wang et al., 2014). Beside the differences between STIM1 and STIM2, the CC regions are highly conserved, presenting, however, noticeable disparity in their functions (Wang et al., 2014).

## Orai Proteins

**FIGURE 3:**
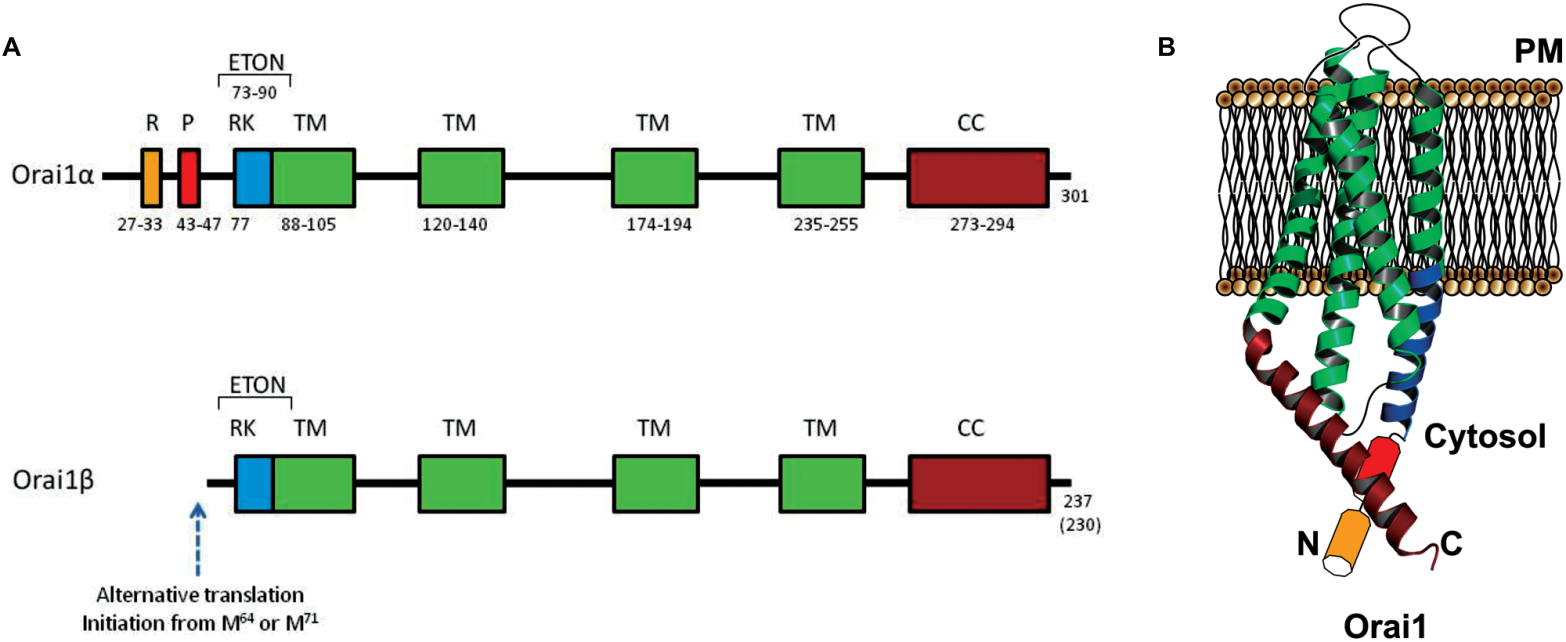
**Molecular structure of Orai1α and Orai1β variants. (A)** Orai1α and Orai1β architecture. R, arginin-rich region; P, proline-rich region; RK, arginine-lysine-rich region; TM, transmembrane domain; CC, coiled-coil domain (Yuan et al., 2009; Derler et al., 2012). Orai1β has an alternative translation initiation from methionine at position 64 or 71 (Fukushima et al., 2012). **(B)** Cartoon depicting Orai1α monomer crystal structure in the resting state.

The Orai channels family is composed by three remarkably conserved homologs: Orai1, Orai2, and Orai3 (Feske et al., 2005; Mercer et al., 2006; Zhang et al., 2006; Gwack et al., 2007; Rothberg et al., 2013). A single Orai1 monomer spams four times the PM, exposing two loops (1 and 3) to the extracellular medium and with the N- and C-terminus domains and one loop (2) facing the cytoplasm (**Figure 3**). Both, N- and C- termini are required for STIM1 interaction and regulation (Muik et al., 2009; Park et al., 2009; Yuan et al., 2009; Derler et al., 2013; Palty et al., 2015; Palty and Isacoff, 2016).

Prior to its crystal structure determination in *Drosophila*, the human Orai1 channel was thought to form a tetramer with high selectivity to Ca^2+^ (Mignen et al., 2008b; Penna et al., 2008; Maruyama et al., 2009). Crystallization of *Drosophila* Orai1 showed a hexameric molecule permeable to Ca^2+^ as well as to monovalent ions in the presence of divalent cations (Hou et al., 2012; Thompson and Shuttleworth, 2013a). Furthermore, the Orai channels may form heteropentamers (3 Orai1 and 2 Orai3) to function as arachidonate-regulated Ca^2+^ (ARC) channels, a store-independent channel regulated by the STIM1 population located in the PM (Thompson and Shuttleworth, 2013b; Zhang et al., 2014).

Regarding CRAC channels, and despite that further studies are needed to determine human Orai1 structure, it is mostly accepted that each Orai1 channel comprises 6 Orai1 monomers, accurately arranged, forming the highly Ca^2+^ selective ion channel in the PM. The pore is located amid the hexamer, involving the six TM1 domains and including the residues 74–90 (ETON region) within the N-terminus (Derler et al., 2013) which contributes to STIM1 binding. Briefly, the pore acts as a funnel formed by the external vestibule, negatively charged (aa D110, D112, and D114) and supposed to attract Ca^2+^ to the immediacies to the pore; next the selectivity filter (aa E106); the hydrophobic cavity (aa V102, F99 and L95) and a basic region (aa R91, K87, and R83). It is surrounded by three rings, subsequently comprised by TM2, TM3, and TM4 (Hou et al., 2012). Even though TM2–4 do not form the pore themselves, it is known that several residues within those, are key regulators of the closed stated of Orai1. For instance, the Orai1 mutations L138F and P245L, located in TM2 and TM4, trigger constitutive currents and have been found to be causing tubular aggregate myopathy (TAM; Endo et al., 2015) and the Stormorken disease (Nesin et al., 2014), respectively. Included into the C-terminus, and in addition to the STIM1-binding region, Orai1 monomers exhibit a highly conserved hinge region, which allows the Orai1 subunits to pair with its neighbor in an antiparallel manner, and to coexist as dimers (Hou et al., 2012).

## STIM1-Orai1 Coupling

STIM1-mediated Orai1 activation has been studied since their partnership was disclosed in 2006. ER Ca^2+^ depletion prompted by a stimulus leads to Ca^2+^ displacement from the STIM1 EF-hand and a number of rearrangements within the N-terminus domain, transferred through the TM domain to the C-termini, which culminates in Orai1 activation and Ca^2+^ entry. Nowadays and despite to the fact that a resting STIM1 crystal structure has proved elusive, it is common consensus that in its coalescent state, STIM1 forms dimers, its EF-hand domain is occupied by Ca^2+^, and that the Orai1 triggering regions are hidden from the channel (Fahrner et al., 2013). While STIM1 response obeys solely to large variations in [Ca^2+^]_ER_, STIM2 shows faster reaction to smaller changes in intraluminal Ca^2+^ (Stathopulos et al., 2009). As stated above, Ca^2+^ dissociation from the EF-hand changes both the later and SAM conformation exposing hydrophobic domains and habilitating the formation of STIM1 dimers and oligomers (Stathopulos et al., 2006, 2009). Next, the TM region, which has been recently shown to interact in an angled manner within the ER membrane, provides support to the control of the active/inactive state of STIM1 dimers. Upon ER depletion the angle lessened bringing the C-termini together (Ma et al., 2015). Ultimately, the signal travels through the molecule to the cytosolic domain where CC1, which is clamping the rest of C-terminal portion in a tight state, releases it in order to reinforce the oligomerizated conformation, via the SHD region, and to grant STIM1 activating regions access to Orai1 C- and N-terminus (Derler et al., 2013; Stathopulos et al., 2013; Fahrner et al., 2014). The interaction between STIM1 C-terminus and Orai1 C-terminus has been recently solved by NMR. In Stathopulos et al. (2013) have demonstrated that the critical positively charged residues mentioned above, K382, K284, K385, and K386, two aromatic ones Y361 and Y362; and finally four hydrophobic amino acids L347, L351, L373, and A376 are the key players within STIM1 to interact with Orai1; meanwhile the channel includes the residues L273, L276, R281, L286, R289 from its C-terminus, forming what the authors have named the STIM1-Orai1 Association Pocket (SOAP; Stathopulos et al., 2013). However, the association between STIM1 and Orai1 N-terminus is still yet completely unsolved and further approaches are required to fully understand the STIM1-mediated gating mechanism of Orai1.

Recent studies have revealed the expression of splice variants of STIM1, STIM2, and Orai1 in different cell types. It has been reported that over 95% multiexonal proteins in vertebrates undergo alternative splicing (Kornblihtt et al., 2013), which expands the functional diversity of a number of genes. Here we present the most prominent STIM1 and Orai1 variants and the differences among them.

## STIM Splicing Variants

### STIM1

As mentioned before, upon cell stimulation, the ER Ca^2+^ concentration decreases and Ca^2+^ unbinds from the canonical EF-hand, leading to an oligomerization of STIM1 molecules followed by a translocation toward the PM and aggregation as punctae structures (Lewis, 2007). Subsequently, STIM1 C-terminal region unfolds, exposing the SOAR domain and locating the polybasic lysine-rich region close to the SOC channels.

Darbellay et al. (2011) an alternatively spliced long variant of STIM1 (STIM1L, L for long to differentiate it from the conventional STIM1 isofom of 90 kDa) was identified in adult human muscle fibers and in *in vitro*–differentiated myotubes. STIM1L was described to be the product of an alternative splicing on exon 11, and contains an extra 106 residues (aa 515–620) inserted in the cytosolic region (**Figure 1**), an actin-binding domain, that allows STIM1L to interact with Orai1 Ca^2+^ channels to form permanent clusters (Darbellay et al., 2011).

While STIM1 is ubiquitously expressed, STIM1L is expressed in human skeletal muscle (Horinouchi et al., 2012), in skeletal muscle, as well as in heart and brain of mice (Darbellay et al., 2011) and in neonatal rat cardiomyocytes (Luo et al., 2012). The expression of STIM1L, as well as that of the conventional STIM1 variant, decreased in adult rat cardiomyocytes, where their expression levels have been found to be upregulated under pathological cardiac hypertrophy (Luo et al., 2012).

The function of STIM1L has been associated to the particularly rapid maximal activation of SOCE in skeletal muscle cells (<1 s) in comparison with other cells where full SOCE activation requires several seconds (>5 s in human platelets (Redondo et al., 2006), and up to 260 s in other cell types (Parekh and Putney, 2005; Liou et al., 2007). STIM1L was initially found to allow rapid activation of SOCE and is required to trigger repetitive cytosolic Ca^2+^ signals (Darbellay et al., 2011). The rapid activation of SOCE in STIM1L expressing cells was proposed to rely on the interaction between STIM1L and Orai1 at rest even when Ca^2+^ stores were full. This interaction was suggested to be stabilized by STIM1L-actin filament association as actin depolymerization has been found to disrupt STIM1L–Orai1 complexes at rest, which, subsequently delays SOCE activation (Darbellay et al., 2011). More recent studies have revealed that both STIM1 and STIM1L are distributed throughout the cortical ER vesicles, while Orai1 channels are localized in the PM. Following agonist stimulation and reduction in ER Ca^2+^ concentration STIM1 induces cortical ER expansion by a mechanism that requires the lysine-rich motif thus recruiting Orai1 channels in large ER–PM clusters. By contrast, STIM1L is unable to enlarge cortical ER structures and recruits Orai1 channels in reduced ER–PM clusters (Sauc et al., 2015). The greater efficiency of STIM1L mediating Ca^2+^ entry through SOCE has been hypothesized to occur due to the slower diffusion of Ca^2+^ in the cytoplasm in large STIM1–Orai1 clusters which might trap Ca^2+^ in the proximity of Orai1 channels promoting Ca^2+^-dependent inactivation of the channel (Sauc et al., 2015).

Furthermore, STIM1L has been reported to modulate store-independent Ca^2+^ entry through the TRPC channels TRPC3 and TRPC6. In HEK-293 cells stably expressing endothelin type A receptor STIM1L expression was found to attenuate receptor-operated Ca^2+^ entry via TRPC3 and TRPC6 more strongly that STIM1 by interaction with both channels (Horinouchi et al., 2012). Overexpression of STIM1 and STIM1L did not modify the expression level of TRPC3 and TRPC6. Although STIM1L exhibits a greater capacity to bind TRPC3 and TRPC6 than STIM1 (Horinouchi et al., 2012), which might suggest the recruitment of these channels into the store-dependent signalplex, the precise mechanism involved in the suppression of store-independent, receptor-operated, Ca^2+^ entry by STIM1 and STIM1L in this cell model has not been further clarified.

### STIM2

The homologue of the STIM1 protein, STIM2 was identified in Williams et al. (2001) as a type I TM protein located in the ER and also identified in acidic organelles (Liou et al., 2005; Zbidi et al., 2011). The human STIM2 gene comprises 13 exons located at 4p15.1 (Williams et al., 2001), which lead to a variety of splice isoforms with different properties.

The best characterized STIM2 isoform is STIM2.2 (Miederer et al., 2015), also known as STIM2α (Rana et al., 2015). Human STIM2.2 consists of 833 amino acid with a molecular weight of 105 (115 kDa for the phosphorylated form), which shares amino acid sequence as well as domain architecture with STIM1 (Lopez et al., 2012). STIM2.2 mRNA is encoded by 12 exons (exons 1–8 and 10–13).

As previously mentioned for STIM1, the N-terminal region of STIM2 is located in the lumen of the ER and comprises a canonical EF-hand motif, a “hidden” EF-hand motif, and a SAM, (Soboloff et al., 2006). The canonical EF-hand motif has the ability to bind Ca^2+^ and exhibits an affinity for Ca^2+^ greater than that of STIM1 (STIM2 EF-hand motif Kd∼0.5 mM, STIM1 EF-hand motif Kd∼0.6 mM; Stathopulos et al., 2006; Zheng et al., 2008), therefore, STIM2 protein shows a greater sensitivity to minor changes in ER Ca^2+^ concentration as compared to STIM1. As a result, STIM2 has been reported to be partially active at resting ER Ca^2+^ concentrations and further actives by small reductions in ER Ca^2+^ concentrations, while STIM1 requires much larger reductions in ER Ca^2+^ concentration, such as those induced by physiological agonists, to become active (Brandman et al., 2007). Such greater sensitivity for free Ca^2+^ confers STIM2 the ability to sense ER Ca^2+^ concentration fluctuations as well as to activate earlier than STIM1 upon agonist-induced ER Ca^2+^ store discharge (Brandman et al., 2007).

The hEF domain, which is unable to bind Ca^2+^, plays an important role in the stability of the canonical EF-hand motif and SAM domains (Stathopulos et al., 2006, 2008; Zheng et al., 2011). On the other hand, the SAM domain plays an essential role in STIM oligomerization (Zheng et al., 2008, 2011).

The C-terminal region of STIM2 is located in the cytosol and comprises an ezrin/radixin/moesin (ERM) domain that contains three CC domains (CC1-3) including the including the SOAR/CAD, which has been reported to bind to Orai and TRPC channels (Yuan et al., 2009; Lee et al., 2014; Prakriya and Lewis, 2015). STIM2 structure differs from STIM1 in the adjacent proline- and histidine-rich (P/H) motif (PHAPHPSHPRHPHHPQHTPHSLPSPDP) located in a position that resembles that of the serine- and proline-rich (S/P) region present in STIM1 (SPSAPPGGSPHLDSSRSHSPSSPDPDTPSP), whose function is still unclear. From this point the sequences of STIM1 and STIM2 are significantly different, except for the distal lysine-rich (K) motif which consists of 14 amino acids in STIM1 (five lysines) and 17 residues in STIM2 (nine lysines; Williams et al., 2001).

The function of STIM2 differs from that of STIM1. While STIM1 is the main activator of SOCE, STIM2 mainly controls the resting cytosolic and ER Ca^2+^ concentrations and modulates prolonged Ca^2+^ entry and response to low concentrations of physiological agonists (Brandman et al., 2007; Oh-Hora et al., 2008). STIM2 is also involved in the regulation of the store-operated *I*_min_ Ca^2+^ channels in HEK-293 cells (Shalygin et al., 2015). In addition, the greater sensitivity of STIM2 to changes in ER Ca^2+^ concentration mentioned above leads to a role for STIM2 in the regulation of Ca^2+^ oscillations that differs from that attributed to STIM1. Thus, silencing of STIM2 expression has been reported to impair agonist-mediated Ca^2+^ oscillations at low levels of store depletion, without interfering with STIM1-mediated Ca^2+^ responses induced by full store discharge (Thiel et al., 2013).

Although the role of STIM2 in the activation of SOCE and its interaction with store-operated channels has been less investigated than that of STIM1, it has been reported that STIM2 can interact functionally with overexpressed as well as endogenously expressed Orai1, -2, and -3 and TRPC1 (Brandman et al., 2007; Parvez et al., 2008; Bandyopadhyay et al., 2011; Zbidi et al., 2011; Berna-Erro et al., 2012; Kar et al., 2012; Stanisz et al., 2014); however, whether STIM2 is relevant for the activation of store-independent Ca^2+^ entry remains unclear.

Two recent studies have identified three STIM2 splice variants: STIM2.1, STIM2.2, and STIM2.3 (Miederer et al., 2015; Rana et al., 2015). The structure and function described above for STIM2 concerns the STIM2.2 variant (**Figure 2**), the conventional isoform of STIM2, which will be named STIM2.2 from now on.

STIM2.1, also known as STIM2β, has been reported to contain an eight-residue insert (VAASYLIQ) in its SOAR/CAD region, encoded by an additional exon 9, that disrupts binding to Orai (Miederer et al., 2015; Rana et al., 2015; **Figure 2**). The expression of the STIM2.1 variant has been reported to be ubiquitous and its abundance relative to STIM2.2 depends upon the cell type but is significantly high in naive T cells, where the expression of both variants is similar (Miederer et al., 2015). In contrast to the role of STIM2.2 as an activator of SOCE, STIM2.1 has been shown to play an inhibitory role. STIM2.1 knockdown increases SOCE in CD4^+^ T cells, while overexpression of STIM2.1 decreases SOCE (Miederer et al., 2015).

The mechanism underlying the inhibitory role of the STIM2.1 variant in SOCE remains unclear. STIM2.1 by itself has been reported to interact poorly with Orai1 as detected by FRET or puncta formation assays (Rana et al., 2015); however, it has been indicated that STIM2.1 might heterodimerize with STIM1 or STIM2.2, which might recruit it to Orai1 channels, increasing the possibility to inhibit SOCE despite its low affinity for the channel. When STIM2.1 is recruited to the Orai1 channel signalplex, it might inhibit SOCE passively by direct interaction with STIM1 or STIM2.2, thus reducing the number of SOAR/CAD domains available for channel activation. However, this passive inhibition is unlikely to play a significant role in the modulation of SOCE under physiological conditions, since STIM2.1 is generally not as highly expressed as STIM1, and, therefore, a stronger active inhibitory role has also been hypothesized (Rana et al., 2015).

The transcript of the third STIM2 variant, STIM2.3, contains an alternative exon 13 that leads to an upstream end of translation and a transcript shortened by 444 bp, which results in a protein with approximately 17 kDa smaller (Miederer et al., 2015; **Figure 2**). The function of the STIM2.3 variant is unknown at present and its expression seems to be quite limited and has not been detected in lymphocytes, where the other two variants are significantly expressed (Miederer et al., 2015).

## Orai1 Splicing Variants

Two variants of Orai1, Orai1α, and Orai1β, have been found to be expressed. Orai1α is the conventional variant of 301 amino acids (∼33 kDa, although the predicted molecular weight might be significantly modified by post-translational modifications, such as glycosylation on the asparagine residue at position 223 (Gwack et al., 2007) or phosphorylation on serine residues at positions 27 and 30 (Kawasaki et al., 2010). The short Orai1 variant, Orai1β, is generated by alternative translation initiation from a methionine at position 64, and possibly also 71, leading to a protein of approximately 23 kDa. Both Orai1 variants have been found to be ubiquitously expressed in human cell lines from a number of tissues, including HEK293 cells, Jurkat T cells, HeLa cells, epidermal HaCaT cells or the T84 lung carcinoma cell line, and show similar cellular localization (Fukushima et al., 2012).

The sequence upstream of the translation initiation of Orai1β includes a proline-rich motif previously suggested to be important for Orai1 gating by STIM1 (Takahashi et al., 2007), and an arginine-rich sequence that has been found to be involved in the interaction of Orai1 with PM phosphatidylinositol-4,5-bisphosphate that might be important for the mobility of Orai1 in the PM (Calloway et al., 2011). In agreement with the latter, Fukushima and coworkers have reported that Orai1β has faster mobility in the PM (Fukushima et al., 2012).

A recent report by Desai et al. (2015) has revealed that both Orai1 variants might be subunits of the store-operated CRAC and SOC channels, with some biophysical differences that includes a stronger Ca^2+^-dependent inactivation of Orai1α. However, the most significant functional difference between these variants lies in the participation of Orai1α, but not Orai1β, in the ARC channels (Desai et al., 2015), although the molecular mechanisms underlying the different biological significance of both variants remain unclear.

Summarizing, different STIM1, STIM2, and Orai1 variants have been reported to be expressed as a result of alternative splicing in a number of cell types. The expression of certain variants, such as STIM1L, is quite restricted while that of other variants is ubiquitous. As it has been hypothesized (Kornblihtt et al., 2013), alternative splicing might expands the functional diversity of multiexonal genes. Consistent with this, significant functional differences have been reported between STIM1 and STIM1L, STIM2.1 and STIM2.2 and between the α and β variants of Orai1, which lead to distinct mechanisms of regulation of Ca^2+^ entry through store-operated (CRAC and SOC) as well as store-independent (ARC) channels. The analysis of the expression ratios of the different variants in a particular cellular model might be of great interest to understand the fine modulation of Ca^2+^ entry.

## Author Contributions

JR designed the manuscript, contribute to write it and performed the final edition. RD, TS, and IJ contributed to write the manuscript and discussion.

## Conflict of Interest Statement

The authors declare that the research was conducted in the absence of any commercial or financial relationships that could be construed as a potential conflict of interest.
